# The complement regulatory protein CD46 serves as a novel biomarker for cervical cancer diagnosis and prognosis evaluation

**DOI:** 10.3389/fimmu.2024.1421778

**Published:** 2024-06-11

**Authors:** Jun-Hui Yu, Hao-Bo Yuan, Zi-Yi Yan, Xia Zhang, Hui-Hui Xu

**Affiliations:** ^1^ Department of Gynecology and Obstetrics, Taizhou Hospital of Zhejiang Province, Wenzhou Medical University, Linhai, Zhejiang, China; ^2^ School of Medicine, Shaoxing University, Shaoxing, Zhejiang, China; ^3^ Medical Research Center, Taizhou Hospital of Zhejiang Province, Wenzhou Medical University, Linhai, Zhejiang, China; ^4^ Biological Resource Center, Taizhou Hospital of Zhejiang Province, Wenzhou Medical University, Linhai, Zhejiang, China

**Keywords:** CD46, complement regulatory protein, cervical cancer, biomarker, prognosis

## Abstract

**Background:**

CD46 has been revealed to be a key factor in malignant transformation and cancer treatment. However, the clinical significance of CD46 in cervical cancer remains unclear, and this study aimed to evaluate its role in cervical cancer diagnosis and prognosis evaluation.

**Methods:**

A total of 180 patients with an initial diagnosis of cervical cancer were enrolled at Taizhou Hospital of Zhejiang Province, China. The plasma levels of soluble CD46 (sCD46) and the expression of membrane-bound CD46 (mCD46) were detected by enzyme-linked immunosorbent assay (ELISA) and immunohistochemistry (IHC), respectively.

**Results:**

CD46 was found to be significantly upregulated in cervical cancer tissues *vs.* normal tissues, while no CD46 staining was detected in paired adjacent noncancerous tissues. CD46 staining was more pronounced in cancer cells than in stromal cells *in situ* (in tissues). Moreover, the plasma levels of sCD46 were able to some extent discriminate between cancer patients and healthy women (AUC=0.6847, 95% CI:0.6152–0.7541). Analysis of Kaplan–Meier survival curves revealed that patients with low CD46 expression had slightly longer overall survival (OS) than patients with high CD46 expression in the tumor microenvironment, but no significant difference. Univariate Cox regression analysis revealed that CD46 (*P*=0.034) is an independent risk factor for OS in cervical cancer patients.

**Conclusion:**

The present study demonstrated that cervical cancer patients exhibit aberrant expression of CD46, which is closely associated with a poor prognosis, suggesting that CD46 plays a key role in promoting cervical carcinogenesis and that CD46 could serve as a promising potential target for precision therapy for cervical cancer.

## 1 Background

Cervical cancer is the 4^th^ most prevalent malignancy worldwide, affecting several hundred thousand women annually. The natural history of cervical cancer is well understood, mainly due to persistent infection with high-risk human papillomavirus (HPV), which causes the majority of all cervical cancers (99%) (1). This cancer is largely considered a preventable disease through early detection of premalignant lesions. Unfortunately, more than 70% of deaths occur in less developed countries that lack well-organized screening and prophylactic vaccination programmes (2). It is well known that cancer treatment depends on the stage at diagnosis; options include surgery, chemotherapy, radiotherapy, targeted therapy, and immunotherapy. Although early-stage cervical cancers can be cured, cases with late metastasis, recurrence and drug resistance remain difficult to treat. In addition, the majority of recurrent or metastatic cervical cancers not amenable to locoregional treatments are considered incurable tumors with a poor prognosis (3). Strikingly, recent advances in immunotherapy have shown promise for these patients, as demonstrated by PD-1/PD-L1 immune checkpoint inhibitors (pembrolizumab, cemiplimab, atezolizumab, avelumab, etc) (4). More importantly, the expression of PD-L1 can be used as a biomarker to predict its therapeutic effect (5). However, the objective response rate (ORR) of patients with PD-L1 positive tumors was only 14.6% (95% CI, 7.8% to 24.2%), indicating that the clinical outcomes of most advanced cancer patients are poor (5). Therefore, there is still an urgent need for more effective biomarkers to accurately determine which patients would receive more benefit from these immunotherapies.

CD46, also known as membrane cofactor protein (MCP), is a type 1 membrane protein that not only protects autologous cells from complement-dependent cytotoxicity (CDC) by inactivating C3b and C4b but also functions as a receptor for certain adenovirus (Ad) and measles virus (MeV) (6–8). Interestingly, it has recently been found that CD46 plays a pivotal role in tumor growth and metastasis. Emerging findings have indicated that overexpression of CD46 in solid cancers such as breast, ovarian, colorectal, and bladder cancers may protect cancer cells from destruction caused by the complement system (8–10). Downregulation of CD46 expression by small interfering RNA (siRNA) could sensitize cancer cells to complement attack *in vitro* (11, 12). Thus, these findings suggest that CD46 could be a favorable target for cancer treatment (8, 13). CD46 may play a key role in the immune response to cancer cells, and elucidating its role in carcinogenesis would be helpful for the treatment of cancer.

In addition, our previous findings based on bioinformatics technology indicated that CD46 might be a key predictor of overall survival in cervical cancer patients that could be used in a prognostic model (14). Consistent with our findings, Chen et al. (15) also suggested that high expression of CD46 was associated with a poor prognosis in cervical cancer patients. However, there is limited knowledge about the role of CD46 in cervical carcinogenesis. In this study, we focused on CD46 expression in plasma specimens, cervical cancer tissues and paired adjacent noncancerous tissues to evaluate the correlation between CD46 and clinical parameters. The findings of this study may provide novel insights into the prognosis of patients with differential expression of CD46 who are receiving immunotherapy for cervical cancer.

## 2 Materials and methods

### 2.1 Study population

From 2008 to 2020, a total of 180 patients (mean: 55.7 years; range: 33~91 years) who were initially diagnosed with cervical cancer at Taizhou Hospital were enrolled (**Table 1**). Among all patients, 165 (91.7%) had squamous cell carcinoma (SCC), 10 (5.6%) had adenocarcinoma (ADC), and 5 (2.8%) had adenosquamous carcinoma (ASC). According to the FIGO classification, there were 58 patients (32.2%) with FIGO stage I, 85 patients (47.2%) with FIGO stage II, 36 patients (20.0%) with FIGO stage III, and 1 patient (0.5%) with FIGO stage IV. A total of 113 patients underwent surgery for lymph node dissection, and 28 (24.8%) had lymph node metastasis. CD46 expression in the cervical microenvironment was detected in 83 cancer tissues and 46 paired adjacent noncancerous tissues. Plasma specimens from 116 patients and 120 unrelated healthy women with no personal or family history of cancer were used to measure soluble CD46 (sCD46) levels. The plasma specimens were separated and stored at -80°C until analysis.

**Table 1:** The clinicopathological characteristics of patients with cervical cancer.

Characteristics	No. (%)
All patients	
	180(100)
Age (years)	
Mean ± SD	55.7 ± 12.9
Range	33 ~ 91
Histological types	
Squamous cell carcinoma (SCC)	165(91.7)
Adenocarcinoma (ADC)	10(5.6)
Adenosquamous carcinoma (ASC)	5(2.8)
FIGO Stage	
I	58(32.2)
II	85(47.2)
III	36(20.0)
IV	1(0.5)
Nodal status	
Negative	85(47.2)
Positive	28(15.6)
Unknown	67(37.2)
Follow-up	
alive	122(67.8)
death	55(30.6)
lost	3(1.7)

### 2.2 Immunohistochemistry and staining evaluation

IHC was performed as previously described (16). Briefly, formalin-fixed and paraffin-embedded tissue sections (4 μm) were dewaxed and rehydrated and then incubated overnight with a monoclonal antibody (clone D6N7H, diluted 1:800) against CD46 (Cat#13241, Cell Signalling) at 4°C. The signals were amplified using the Dako EnVision kit (Cat#GK500705, Dako) for visualization of the immunohistochemical reaction.

CD46 staining was independently evaluated by two pathologists who were unaware of the clinical information of these patients. If the percentage of cells with CD46 expression (indicated by staining), was less than 5%, expression was considered negative; positive CD46 staining was scored as follows: 1+ (6–25%), 2+ (26–50%), 3+ (51–75%), and 4+ (>75%).

### 2.3 sCD46 enzyme-linked immunosorbent assay

The concentration of sCD46 (pg/mL) was measured using an ELISA kit (Cat# EH1452, Wuhan Fine Biotech, China), and the absorbance was measured at 450 nm. Then, according to the manufacturer’s instruction, the final concentration (range: 7.812–500 pg/mL) was determined by optical density based on an eight-point calibration curve.

### 2.4 Statistical analysis

For statistical analysis, we used SPSS 21.0 (SPSS, Inc., Chicago, IL, USA) and GraphPad Prism 8.0 (GraphPad Inc., San Diego, CA). *P* < 0.05 (two-tailed) was considered to indicate statistical significance. Correlations between CD46 expression and clinical parameters were assessed using a nonparametric test. The feasibility of using plasma sCD46 as a potential biomarker for distinguishing patients with cervical cancer was assessed using receiver operating characteristic (ROC) curve analysis. The areas under the ROC curves (AUCs) were calculated and subjected to statistical analysis. Kaplan−Meier plotter analysis and the log-rank test were performed for survival analysis. Cox regression analysis was used to evaluate the associations between survival and clinical parameters. Overall survival (OS) time was calculated from the date of diagnosis to the date of death or to the last follow‐up (February 26, 2024).

## 3 Results

### 3.1 CD46 expression in cervical cancer tissues

**Figure 1:**
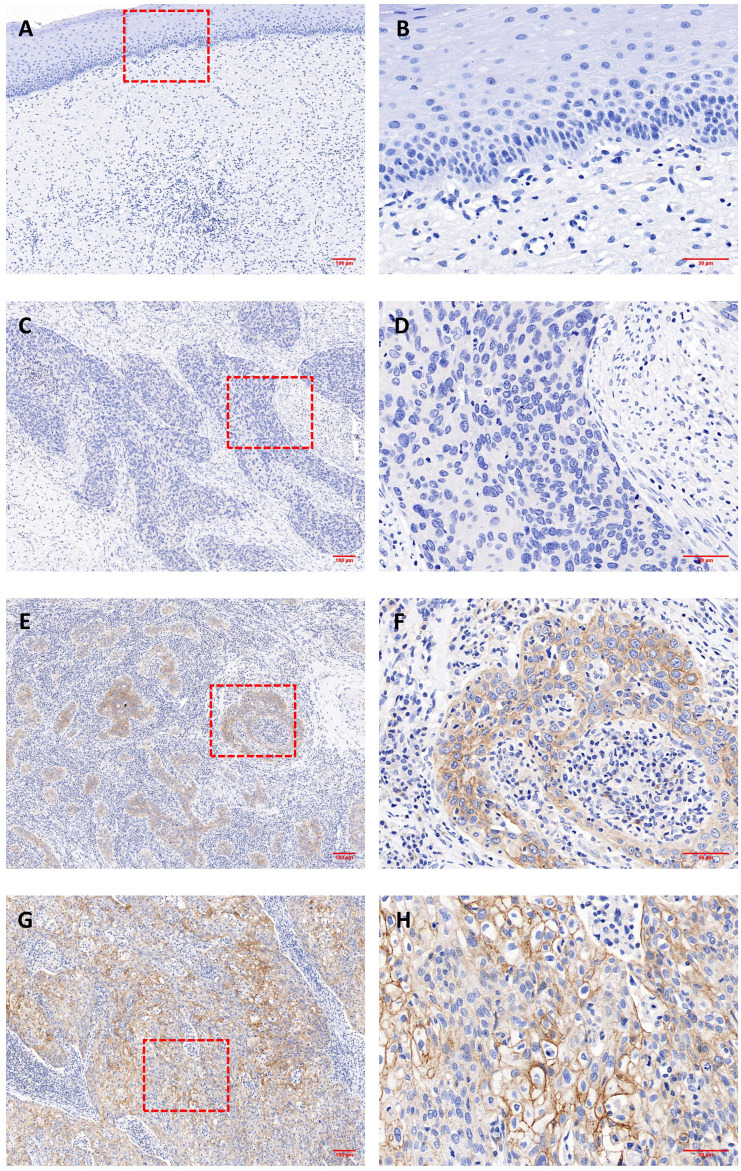
Immunohistochemical analysis of CD46 expression in cervical cancer and paired adjacent noncancerous tissues. **(A, B)** negative CD46 staining in noncancerous cervical tissues; **(C-H)** cervical cancer tissues; Representative staining of negative **(C, D)** and positive expression (**E–H**) of CD46 in cervical cancer lesions. Original magnification: **(A, C, E, G)** (100×) and **(B, D, F, H)** (400×).

Representative IHC images are shown in **Figure 1**, and positive staining was observed in both the cell membrane and cytoplasm. In the following text, the mCD46 positive staining mentioned usually involves membrane and cytoplasm. Overall, 86.7% (72/83) of the primary cervical cancer tissues were mCD46 positive. As depicted in **Figure 1**, heterogeneous staining of mCD46 was detected in cervical cancer tissues, and its expression in cervical lesions ranged from negative to 98%. In the tumor microenvironment (TME), mCD46 staining was more pronounced in cancer cells than in stromal cells. No mCD46 staining was detected in paired adjacent noncancerous tissues. The associations of mCD46 expression in cervical lesions with clinical parameters are summarized in **Table 2**. High mCD46 expression was significantly associated with lymph node metastasis (*P* < 0.001) and survival (*P* < 0.001) in patients.

**Table 2:** The association between CD46 expression and clinical parameters in tumor microenvironment.

Characteristics	No. (%)	CD46 expression					*P* value
		Neg. (<5%)	1+ (6-25%)	2+(26-50%)	3+(51-75%)	4+(>75%)	
Histological types							
SCC	75	11	23	9	14	18	<0.001
ADC + ASC	8	0	3	1	2	2	
FIGO Stage							
I	39	7	14	4	6	8	0.659
II	43	4	12	6	9	12	
Nodal status							
Negative	58	8	19	7	12	12	<0.001
Positive	20	3	6	3	1	7	
Follow-up							
alive	58	9	20	8	9	12	<0.001
death	22	2	6	2	5	7	

P value calculated from Chi-square (χ^2^) test.

### 3.2 Plasma sCD46 levels in patients

**Figure 2:**
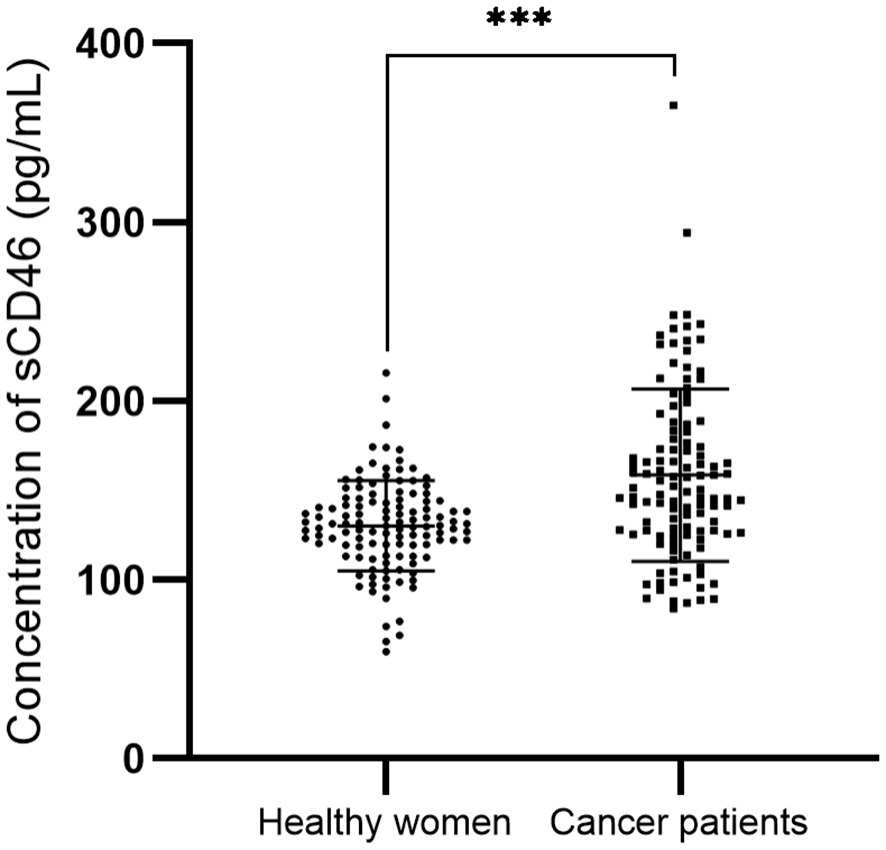
The sCD46 plasma levels between healthy women and cervical cancer. ***P<0.001.

In cervical cancer patients, the peripheral sCD46 levels (mean: 158.4 pg/ml; range: 84.1~365.3 pg/ml) were significantly greater than those in healthy women (mean: 130.2 pg/ml; range: 59.7~215.7 pg/ml; *P* < 0.001) (**Figure 2**). However, the sCD46 level was not associated with FIGO stage, nodal metastasis status, or survival status (**Table 3**).

**Table 3:** The association between the peripheral sCD46 levels and clinical parameters.

Characteristics	Median of molecules values (25-75th)	*P* value
Histological types		
SCC	146.9(125.8-176.0)	0.077
ADC + ASC	188.1(150.5-232.2)	
FIGO Stage		
I + II	149.5(128.5-175.3)	0.903
III + IV	149.1(127.4-187.2)	
Nodal status		
Negative	161.0(132.2-188.8)	0.287
Positive	142.9(121.8-167.0)	
Follow-up		
alive	158.1(127.6-185.8)	0.316
death	143.7(125.1-178.2)	

P value calculated from Mann-Whitney *U* Test.

### 3.3 ROC analysis for sCD46 as a biomarker

**Figure 3:**
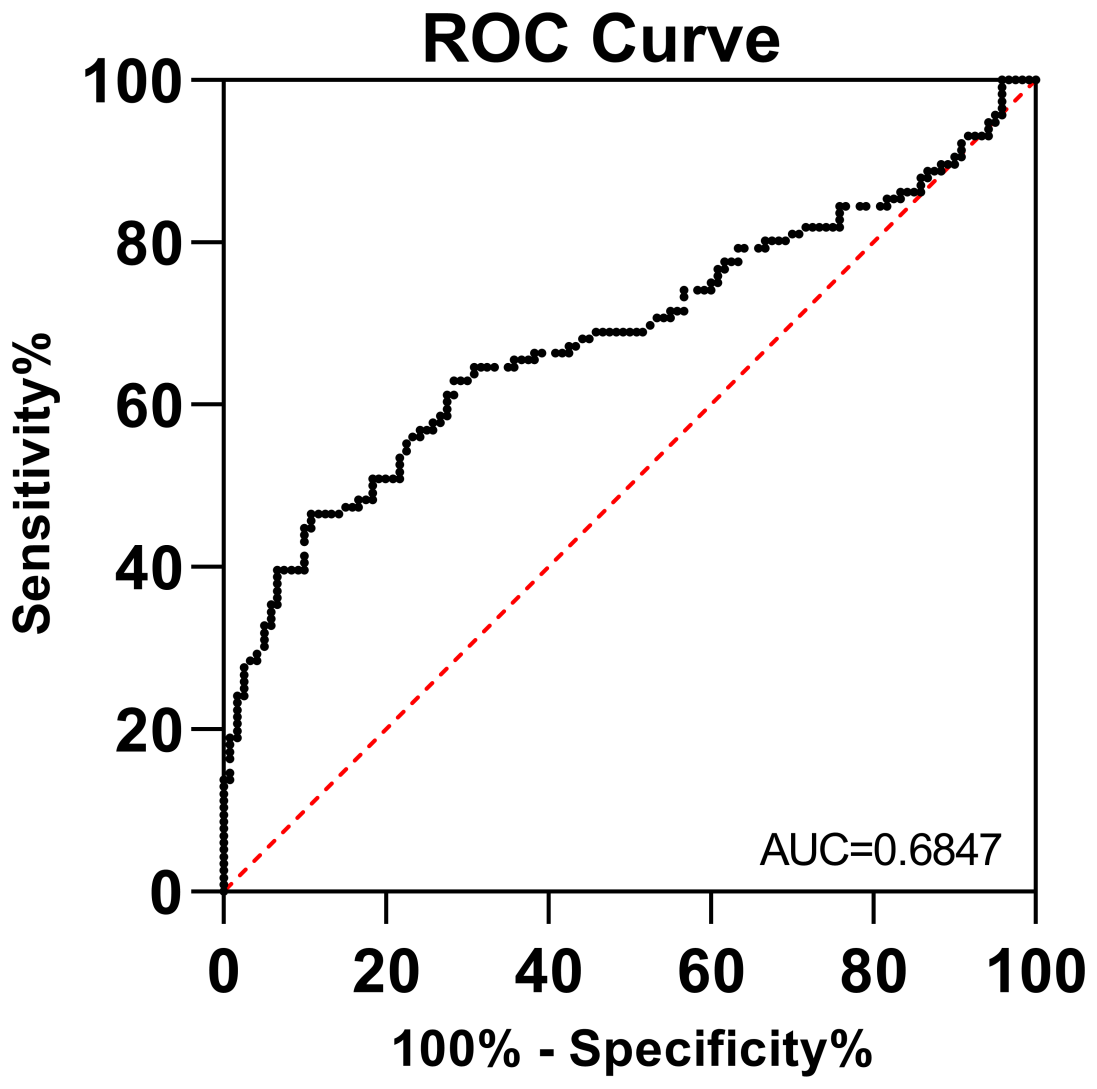
ROC analysis of sCD46 between cervical cancer patients and healthy women. The AUC was 0.6899 (95% CI: 0.6129–0.7668).

To evaluate whether sCD46 could discriminate cervical cancer patients from healthy women, we performed receiver operating characteristic (ROC) analysis. As depicted in **Figure 3**, the ROC curve for sCD46 showed an area under the curve (AUC) of 0.6847 (95% CI: 0.6152–0.7541). The results showed that plasma levels of sCD46 were able to some extent discriminate between cancer patients and healthy women, suggesting that sCD46 could be used as a potential biomarker for the diagnosis of cervical cancer.

### 3.4 Survival analysis

**Figure 4:**
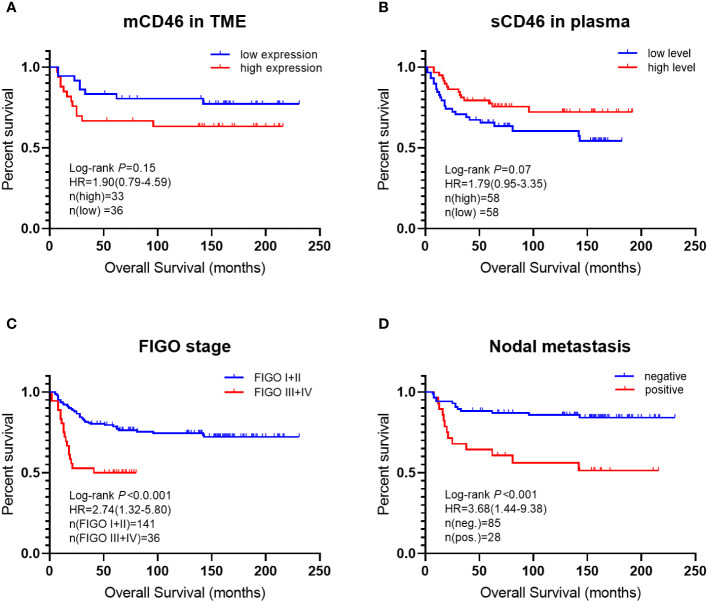
Kaplan–Meier curves of overall survival for cervical cancer patients. differences in overall survival time between: **(A)** low CD46 expression and high CD46 expression in TME; **(B)** low sCD46 levels and high sCD46 levels in plasma; **(C)** FIGO stage I + II and stage III + IV; **(D)** nodal metastasis positive and negative.

The patients were followed for 19 years (mean: 98.2 months; range: 2~231 months) or until death. Among them, 55 patients (30.6%) died of the disease, and 3 (1.7%) were lost to follow-up. Kaplan–Meier analysis of clinical parameters for patient survival is shown in **Figure 4**. The results showed that the OS time of patients with high mCD46 expression was slightly shorter than that of patients with low mCD46 expression in the TME. However, we found the opposite results for sCD46 in peripheral plasma, which showed that the OS time of patients with high sCD46 levels was slightly longer than that of patients with low sCD46 levels. However, there were no significant difference (*P*>0.05). In addition, univariate Cox regression analysis revealed that mCD46 (*P*=0.034), FIGO stage (*P*=0.035), and lymph node metastasis (*P*=0.008) were found to be independent risk factors for OS.

Then, we analyzed the RNA-seq data from the TCGA using the GEPIA platform (http://gepia.cancer-pku.cn/index.htm) to support our findings. The data suggested that cervical cancer patients with high CD46 expression had significantly shorter OS than patients with low CD46 expression (*P*<0.05) (**Supplementary Figure S1**).

## 4 Discussion

The complement system is an evolutionarily ancient response system that is involved in innate immune responses against invading pathogens and damaged cells, including cancer cells. Many studies have demonstrated that the complement system also participates in adaptive immunity, thrombotic disorders, autoimmune disorders, and tumor development in addition to its role in innate immunity. Complement regulatory proteins (CRPs) (i.e., CD46, CD55, and CD59) are expressed in each cell with different expression patterns and mainly play an inhibitory role in preventing overactivation of the complement system. However, these CRPs can also overregulate the complement system, preventing it from eliminating cancer cells (13).

In this study, we showed that CD46 was significantly upregulated in cervical cancer tissues, while the soluble CD46 level was significantly greater in cervical cancer tissues than in healthy tissues. Consistent with our findings, higher expression of CD46 has been observed in ovarian cancer, breast cancer, prostate cancer, bladder cancer, and colon cancer tissue *vs.* adjacent normal tissues (13, 17–21), and the levels of sCD46 have also been reported to be increased (22). In addition, we suggest that CD46 expression could serve as an independent risk factor for overall survival in cervical cancer patients, while sCD46 could be used as a potential biomarker for the diagnosis of cervical cancer. Similar findings have been reported in other studies of gynecological tumors, in which CD46 expression was related to poor prognosis in ovarian and breast cancer patients and served as an independent risk factor for survival (17, 18). Nguyen et al. (19) reported that CD46 plays a key role in promoting the migration of colon cancer cells in TME and can be used for molecular staging and diagnosis. Interestingly, Khan et al. (23) reported that CD46 was more highly expressed in HPV-positive (SiHa and HeLa) cervical cancer cell lines than in HPV-negative (C33A) cervical cancer cell lines and may serve as an early diagnostic marker for HPV-driven cervical carcinogenesis. In addition, CD46 expression may be a survival pathway for cervical cancer cells to escape from tumor-specific CDC. Therefore, our research findings strongly support the association between CD46 expression and cervical cancer development.

Notably, the functional activity of CD46 is not restricted to the cell membrane. A portion of functionally active soluble CD46 is constitutively released from the cell membrane surface into body fluids by endogenous matrix metalloproteinases (MMPs) (24, 25). sCD46 has been detected in body fluids such as plasma, tears, and most notably in seminal fluid (26). The expression of CD46 is stringently regulated by selective splicing, tissue-specific and malignancy-related factors. Several forms of sCD46 have been detected *in vivo*, with molecular masses of 29, 47, and 56 kDa, respectively. Another full-length CD46 (60–65 kDa) was found on vesicles in an intact form, which can be solubilized into the culture medium by metalloproteinases (24). It was found that the 29 kDa sCD46 probably released from the membrane by proteolytic cleavage of mCD46, and the 47 kDa and 56 kDa sCD46 which can be produced by intron retention of mRNA (27). A previous study shows that the 47 kDa and 56 kDa sCD46 are particularly increased in cancer patients sera (22). In this study, we found that total sCD46 plasma levels were able to discriminate between cervical cancer patients and healthy women but were not associated with FIGO stage or with lymph node metastasis. In addition, Pearson correlation coefficient analysis revealed no correlations between sCD46 and cytokines (IL-2, IL-6, IL-10, IFN-γ, etc.) (**Supplementary Figure S2**). As is well known, malignant transformation of cells is accompanied by changes in the surrounding stroma. The shedding of surface molecules on cancer cell membranes may promote endothelial cell migration, invasion, tumor angiogenesis, and immune escape (28). We speculate that in the early stages of cervical cancer, the secretion of sCD46 may mainly derived from the shedding of mCD46, which plays a role in promoting the inactivation of C3b and C4b in TME. However, the role of sCD46 forms in the development of cervical cancer, and its mechanism of action remains unknown.

Currently, targeting CD46 as a therapeutic strategy in cancers has attracted increasing attention to overcome the poor therapeutic efficacy of conventional cancer treatments, including the inhibition of CD46 expression (11, 29–31), neutralizing (blocking) mAbs (30–32), anti-CD46 antibody−drug conjugates (CD46-ADCs) (21, 33–36), and oncolytic virotherapy (37–42). It has been demonstrated that targeted downregulation of CD46 expression by siRNA *in vitro* increases the sensitivity of cancer cells to CDC (11). In a murine model of metastatic bladder cancer, targeted downregulation of Crry (the murine counterpart of CD46) induced a protective antitumor CD8+ T-cell response (29). Furthermore, after therapeutic monoclonal antibody (mAb) treatment, downregulation of CD46 expression in cancer cells could enhance the CDC effect and improve therapeutic outcomes (30, 31). Notably, antibody-dependent cellular cytotoxicity (ADCC) is the major mechanism of therapeutic antibody stimulation. Do et al. (32) reported that treatment of bladder cancer cells with cetuximab inhibited CD46 expression and subsequently promoted both CDC and ADCC, which might be a beneficial mechanism of mAb immunotherapy for cancer treatment.

CD46-ADCs are novel compounds consisting of cytotoxic agents linked to the CD46 antibody that are able to specifically recognize CD46 expressed on the surface of cancer cells (33). Sherbenou et al. (34) suggested that CD46-ADC has the potential to be an effective treatment for multiple myeloma (MM), especially in patients with a gain of chromosome 1q. The curative potential of CD46-ADC has been confirmed in a patient-derived xenograft (PDX) model (35). In addition, exploratory toxicology studies of CD46-ADC in nonhuman primates have demonstrated an acceptable safety profile (21). A phase I clinical trial of CD46-ADC (FOR46, Fortis Therapeutics) has been completed in patients with relapsed or refractory MM (NCT03650491), showing single-agent activity with a partial response or better in approximately one-third of patients. This agent has also been used for the clinical treatment of metastatic prostate cancer by targeting CD46 (NCT03575819) (21, 33). In combination with these findings and our previous studies in cervical cancer, the design of the present study suggest that CD46 may be an excellent target for antibody-based therapy development in cancers.

Additionally, oncolytic virotherapy is a promising immunotherapy against cancer that can target and kill cancer cells and even stimulate immunotherapeutic effects in patients. Oncolytic MeV- and Ad5-based vectors (targeting CD46) have been exploited as popular vectors for cancer therapeutic applications, including vaccines (37, 41). In a series of studies, it was shown that targeting CD46 chimeric Ad5/35 vectors could increase antitumor activity, decrease liver toxicity, and improve the safety profile of treatment for cervical (38), colorectal (39, 42) and bladder cancers (40), especially low-risk bladder cancer. It is worth noting that despite the increasing number of cancer therapeutic approaches using these chimeric vectors, CD46 expression in various cancer types needs to be assessed in individual patients to predict the immune response and patient outcomes.

In conclusion, we showed that CD46 is generally overexpressed in cervical cancer tissues and that high CD46 expression, as determined by IHC, predicts a poor prognosis in patients with cervical cancer. These findings suggest that CD46 plays a unique role in tumorigenesis and could serve as a promising target for precision therapy for cervical cancer. The limitation of this study is that it did not further explore the mechanism of CD46 in cervical carcinogenesis, as well as the potential biological functions of soluble CD46. Therefore, further detailed research in our future studies are needed to clarify the mechanism of action of CD46, and its application in cancer treatment.

## Data availability statement

The original contributions presented in the study are included in the article/**Supplementary Material**. Further inquiries can be directed to the corresponding author.

## Ethics statement

The studies involving humans were approved by Medical Ethics Review Committee of Taizhou Hospital. The studies were conducted in accordance with the local legislation and institutional requirements. The human samples used in this study were acquired from primarily isolated as part of your previous study for which ethical approval was obtained. Written informed consent for participation was not required from the participants or the participants’ legal guardians/next of kin in accordance with the national legislation and institutional requirements.

## Author contributions

JY: Formal analysis, Writing – original draft. HY: Data curation, Methodology, Software, Writing – original draft. ZY: Investigation, Validation, Writing – review & editing. XZ: Methodology, Software, Writing – original draft. HX: Conceptualization, Data curation, Funding acquisition, Writing – original draft, Writing – review & editing.

## Supplementary material

Supplementary Figure 1 The prognostic values of CD46 expression on survival in cervical cancer patients (Kaplan-Meier Plotter). **(A)** overall survival; **(B)** disease-free survival; Survival curves for high (red) and low (blue) expression groups dichotomized at the optimal cutpoint are plotted. The X-axis represents time and the Y-axis represents survival rate.

Supplementary Figure 2 Pearson correlation between sCD46 and cytokines in cervical cancer patients (n=116).
