# Supplementary figures and images for: The complement regulatory protein CD46 serves as a novel biomarker for cervical cancer diagnosis and prognosis evaluation
Source: Front Immunol. 2024 Jun 11;15:1421778. doi: 10.3389/fimmu.2024.1421778 (PMC11196419; doi:10.3389/fimmu.2024.1421778)

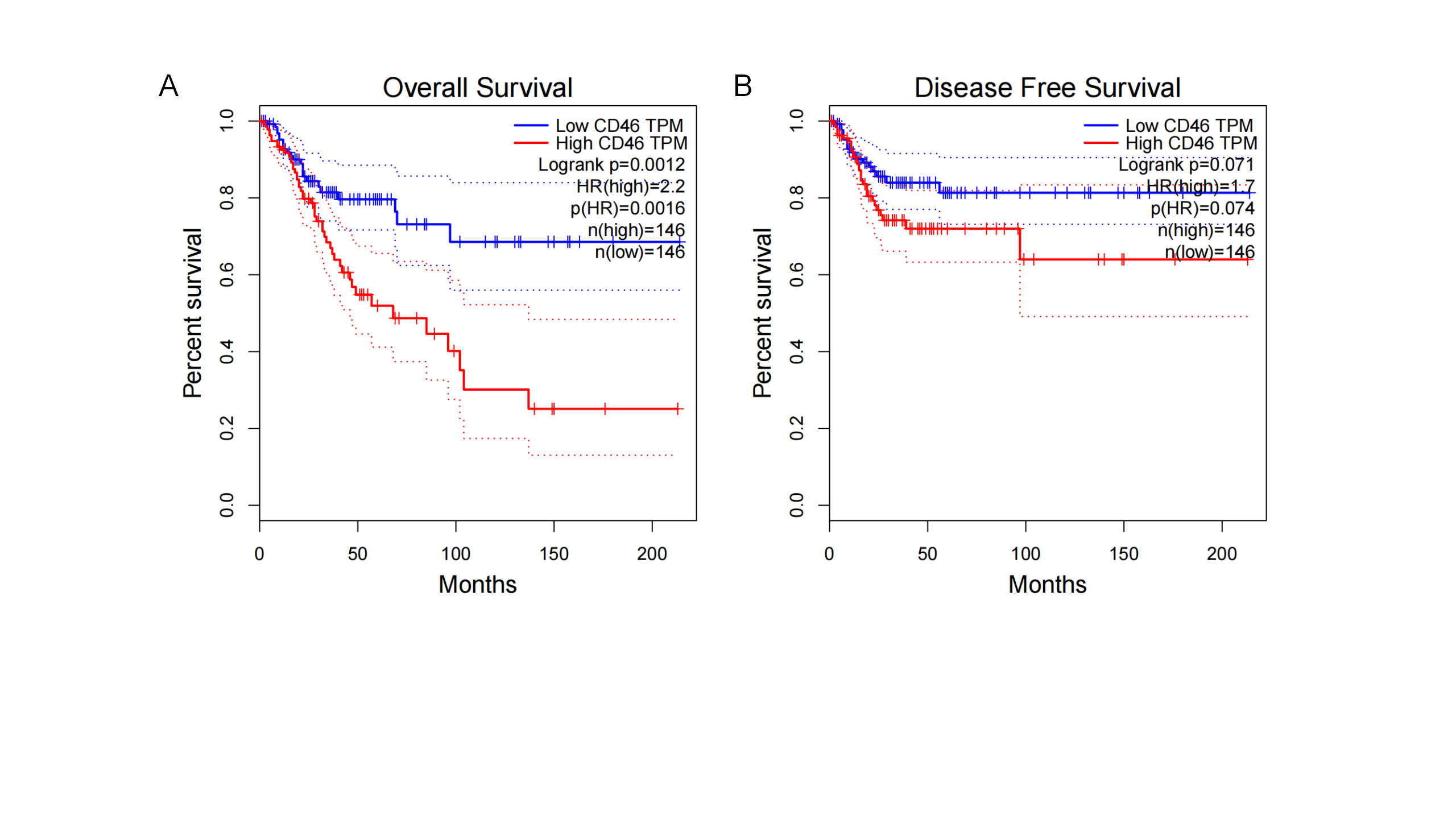

Supplement: Supplementary Figure 1 — The prognostic values of CD46 expression on survival in cervical cancer patients (Kaplan-Meier Plotter). (A) overall survival; (B) disease-free survival; Survival curves for high (red) and low (blue) expression groups dichotomized at the optimal cutpoint are plotted. The X-axis represents time and the Y-axis represents survival rate. [file Image_1.tif]

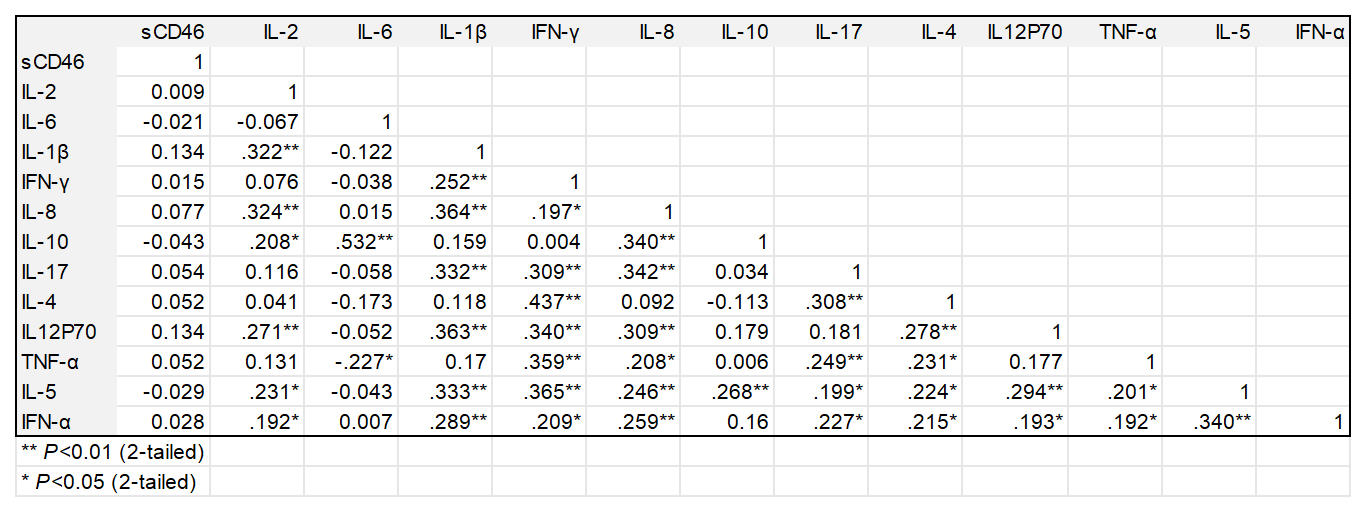

Supplement: Supplementary Figure 2 — Pearson correlation between sCD46 and cytokines in cervical cancer patients (n=116). [file Image_2.tif]
